# The ferroptosis and iron-metabolism signature robustly predicts clinical diagnosis, prognosis and immune microenvironment for hepatocellular carcinoma

**DOI:** 10.1186/s12964-020-00663-1

**Published:** 2020-10-28

**Authors:** Bufu Tang, Jinyu Zhu, Jie Li, Kai Fan, Yang Gao, Shimiao Cheng, Chunli Kong, Liyun Zheng, Fazong Wu, Qiaoyou Weng, Chenying Lu, Jiansong Ji

**Affiliations:** 1 Key Laboratory of Imaging Diagnosis and Minimally Invasive Intervention Research, Lishui Hospital, School of Medicine, Zhejiang University, Lishui, 323000 China; 2 Department of Radiology, Second Affiliated Hospital, School of Medicine, Zhejiang University, Hangzhou, 310058 China; 3 Department of Radiology, the Fifth Affiliated Hospital of Wenzhou Medical University, Lishui, 323000 China

**Keywords:** Ferroptosis, Hepatocellular carcinoma (HCC), TMB, Immune microenvironment, Prognosis

## Abstract

**Background:**

In this study, we comprehensively analyzed genes related to ferroptosis and iron metabolism to construct diagnostic and prognostic models and explore the relationship with the immune microenvironment in HCC.

**Methods:**

Integrated analysis, cox regression and the least absolute shrinkage and selection operator (LASSO) method of 104 ferroptosis- and iron metabolism-related genes and HCC-related RNA sequencing were performed to identify HCC-related ferroptosis and iron metabolism genes.

**Results:**

Four genes (ABCB6, FLVCR1, SLC48A1 and SLC7A11) were identified to construct prognostic and diagnostic models. Poorer overall survival (OS) was exhibited in the high-risk group than that in the low-risk group in both the training cohort (*P* < 0.001, HR = 0.27) and test cohort (P < 0.001, HR = 0.27). The diagnostic models successfully distinguished HCC from normal samples and proliferative nodule samples. Compared with low-risk groups, high-risk groups had higher TMB; higher fractions of macrophages, follicular helper T cells, memory B cells, and neutrophils; and exhibited higher expression of CD83, B7H3, OX40 and CD134L. As an inducer of ferroptosis, erastin inhibited HCC cell proliferation and progression, and it was showed to affect Th17 cell differentiation and IL-17 signaling pathway through bioinformatics analysis, indicating it a potential agent of cancer immunotherapy.

**Conclusions:**

The prognostic and diagnostic models based on the four genes indicated superior diagnostic and predictive performance, indicating new possibilities for individualized treatment of HCC patients.

Video Abstract

**Graphical abstract:**

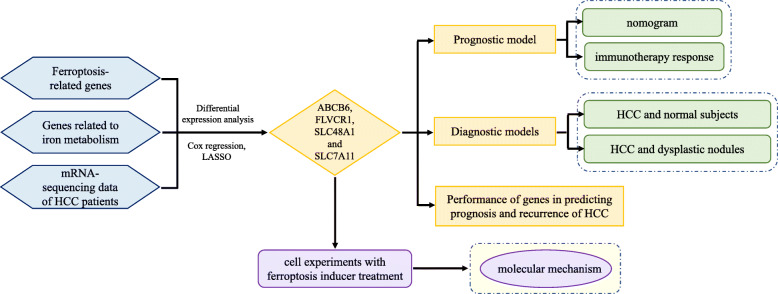

**Supplementary information:**

The online version contais supplementary material available at 10.1186/s12964-020-00663-1.

## Background

As the most frequent primary malignant tumor of the liver [1], hepatocellular carcinoma (HCC) is ranked as the sixth most commonly diagnosed neoplasm and is estimated to be the fourth leading cause of cancer-related death worldwide [2]. The incidence and mortality of HCC are continuing to increase [3]. Based on multiple staging systems, HCC has made great progress in diagnosis and treatment, but most HCC staging systems are currently based on tumor burden and stratification of the disease by prognosis [4]. These systems lack sensitivity and have difficulty explaining the adverse biological characteristics that affect treatment and survival response, which generally limits the treatment effect for patients [5]. HCC is a highly complex, multistep process involving genetic mutations, chromosomal aberrations, molecular signaling pathways, and epigenetic disorders [6]. Therefore, a better understanding of the molecular changes, molecular mechanisms and characterization involved in tumorigenesis and the identification of novel biomarkers that can individually predict the diagnosis and prognosis of tumors are essential for personalized medicine [7].

Iron is a basic nutrient element in the human body, and it is indispensable for biological processes such as cell metabolism, growth and proliferation [8]. The homeostasis of iron metabolism is stably regulated through balanced absorption, systemic transport, and cellular uptake and storage [9]. Alterations in iron metabolism have a dual effect on tumor cells. Since most tumor cells have an increased demand for iron, an increase in iron reserves within a certain range can promote the growth and proliferation of tumor cells [10, 11], and there is a positive correlation between the risk of tumors and iron accumulation [12, 13], but the excessive increase in iron concentration in the body leads to cell death caused by membrane lipid peroxidation, termed ferroptosis [14]. Ferroptosis has anticancer functions that are useful in cancer treatment [15]. Since the first demonstration in 2012, ferroptosis has received widespread attention as a potential therapeutic pathway for cancer treatment. Various studies have determined the key role of ferroptosis in killing tumor cells and inhibiting tumor growth [16, 17]. Some previous studies have also confirmed the important significance of ferroptosis for the treatment and prognosis of liver cancer [18, 19], but the detailed signal transduction pathways and key regulators of ferroptosis during the occurrence and progression of HCC are unclear.

As an emerging feature of cancer, TMB was first emphasized in next-generation sequencing analysis [20]. TMB is defined as the total number of somatic coding mutations associated with the emergence of new antigens that trigger antitumor immunity [21]. It is speculated that highly mutated tumors are more likely to carry neoantigens, making them targets for activated immune cells [22]. Currently, immune checkpoint inhibitor-based immunotherapy as an innovative therapy for multiple types of advanced cancer is emerging, and TMB has been identified as an emerging biomarker that is sensitive to immune checkpoint inhibitors [23]. TMB can help identify patients with some types of cancer that could benefit from immunotherapy.

Tumor immune microenvironment (TIME) mainly refers to immune cells and immune related molecules in the tumor microenvironment. TIME plays a vital role in controlling iron metabolism and homeostasis [24]. Many cell types, such as Th1 cells, natural killer T (NKT) cells, monocytes and macrophages, are involved in the maintenance of iron homeostasis [25]. In addition, ferroptosis was found to work synergistically with immunoregulation in TIME. The lethal ferroptosis in tumor cells can expose tumor antigens, thereby improving the immunogenicity of the microenvironment and enhancing the effectiveness of immunotherapy [26]. And a study also confirmed that immunotherapy can activate CD8 + T cells in the TIME to enhance the lipid peroxidation specific to ferroptosis in tumor cells, and the increase in ferroptosis further promotes the anti-tumor efficacy of immunotherapy [27].

In this study, we used high-throughput methodology technology to comprehensively analyze the genome of HCC and thousands of molecular targets, identify iron metabolism and ferroptosis-related genes closely associated with the prognosis of HCC, construct predictive models for the diagnosis and prognosis of HCC, and explore the relationship with immune infiltration in HCC. Our findings may help improve the early diagnosis rate of HCC and further improve the clinical outcomes of patients under personalized treatment.

## Methods

### Acquisition of ferroptosis- and iron metabolism-related genes associated with HCC

Ferroptosis-related genes were obtained in the ferroptosis pathway (map04216) from the KEGG PATHWAY Database (https://www.genome.jp/kegg/pathway.html). Genes related to iron metabolism were obtained in the pathway of iron uptake and transport (R-HSA-917937) from the Reactome Pathway Database (https://reactome.org/) and cellular iron ion homeostasis (GO:0006879) from the AmiGo2 database (http://amigo.geneontology.org/amigo) [28]. We searched and comprehensively analyzed the source literature of these genes, eliminated unrelated genes and added newly reported related genes, and then integrated them for subsequent research.

### Identifying differentially expressed genes (DEGs) between HCC and adjacent nontumor tissues

The mRNA-sequencing data of patients with HCC with clinical information were downloaded from the TCGA database (including 370 HCC tissue samples and 50 normal tissue samples for a total of 10,000 encoding mRNA sequences) and the ICGC database (including 202 normal samples and 243 HCC samples for a total of 19,677 encoding mRNA sequences). Matching the mRNA-sequencing data with ferroptosis- and iron metabolism-related genes and using limma, an R package, with an absolute log2-fold change (FC) > 1 and an adjusted *P* value < 0.05 to perform differential expression analysis, DEGs related to ferroptosis and iron metabolism were thereby identified. Since the data of the TCGA database and the ICGC database are open to the public and can be downloaded freely, and this study strictly followed the publication guidelines and access policies of the databases, ethical review and approval from an Ethics Committee are not required for the study.

### Establishment and validation of a prognostic predictive signature

Using univariate Cox regression analysis to screen out genes related to OS in patients with HCC, genes with a P value < 0.05 were considered statistically significant and incorporated into the subsequent LASSO Cox regression. In the LASSO-penalized Cox regression analysis, we adjusted the L1 penalty parameter via 10-fold cross-validation to narrow the number of genes, and genes that appeared with a repetition frequency greater than 900 times in 1000 substitution samplings were considered to be more closely related to OS. Based on a multivariate Cox regression for these genes, we built a prognostic signature. The prognostic risk score was determined using a linear combination of the regression coefficient (β) in a multivariate Cox regression model and the expression levels of the genes. Prognostic index (PI) = (β * expression level of ABCB6) + (β * expression level of FLVCR1) + (β * expression level of SLC48A1) + (β * expression level of SLC7A11). X-tile software [29] was used to determine the optimal cut-off value, which help divide patients with HCC into high-risk group and low-risk group and show the most significant difference in prognosis between two groups. Kaplan-Meier (K-M) survival curves and time-dependent receptor operating characteristic (ROC) curves were performed to evaluate the predictive performance of the prognostic signature on OS.

### Independence of the prognostic signature from traditional clinical characteristics

Univariate and multivariate Cox regression analyses were performed to confirm whether the prognostic signature was independent of other traditional clinical characteristics (including age, AFP, weight, vascular tumor cell, sex, pathological grade and TNM stage) in predicting OS of patients with HCC. Hazard ratios (HRs) and 95% confidence intervals (CIs) for each variable were calculated. *P* < 0.05 was considered statistically significant.

### Construction and evaluation of a predictive nomogram

We integrated the independent predictive factors identified by multivariate Cox regression and constructed a predictive nomogram and corresponding calibration maps using “rms” R software. Calibration and discrimination were carried out to validate the calibration maps. The consistency index (C Index), which was calculated via a bootstrap method with 1000 resamples, was used to evaluate the prediction accuracy of the nomogram compared to the actual result and to graphically plot the actual observed rate and the predicted rate of the nomogram to evaluate the calibration curves. The closer the calibration curve is to the 45° line, which represents the best prediction, the better is the prognostic prediction performance of the nomogram. ROC curve analysis was performed to validate the sensitivity and specificity of the nomogram compared to a single independent predictor in predicting OS, and decision curve analysis (DCA) was performed to evaluate the clinical benefit that the nomogram can obtain compared to a single independent prognostic predictor. *P* < 0.05 was considered statistically significant.

### Internal and external validation of the expression characteristics of ferroptosis- and iron metabolism-related genes

Wilcoxon signed rank tests in Prism 7.0 (GraphPad, San Diego, CA, USA) were used to validate the expression characteristics of ferroptosis- and iron metabolism-related genes between HCC and normal tissues in the HCC cohort from ICGC and GSE6764. P < 0.05 was considered statistically significant. Regression analysis was performed to explore the correction among the expression profiles of genes. ROC curve analysis was performed to validate the predictive ability of the genes for OS.

### Estimation of immune cell type fractions

Cell-type Identification By Estimating Relative Subsets Of RNA Transcripts (CIBERSORT) analysis was used to quantitatively convert the transcriptome data of tumor tissue into the absolute abundance of immune cells and stromal cells to assess the proportion of 22 human immune cell subpopulations, including seven T cell types, naïve and memory B cells, plasma cells, NK cells, and myeloid subsets [30, 31]. Standard annotation files were adopted to organize gene expression characteristics. The R package “CIBERSORT” was applied to convert mRNA data into the infiltration fractions of non-tumor cells in the tumor microenvironment. For each sample, the sum of all estimated immune cell type scores is equal to 1.

### Cell culture

Human HCC cell lines (SK-HEP1 and SMMC-7721) were purchased from American Type Culture Collection (ATCC) (Manassas, VA, USA). The cell lines were cultured in DMEM (Gibco, NYC, USA) supplemented with 10% heat-inactivated fetal bovine serum (Gibco, NYC, USA) at 37 °C and maintained in a humidified cell incubator with an atmosphere of 5% CO2.

### Cell viability assay

Cell viability was measured by Cell Counting Kit-8 assay (CCK-8) (Dojindo, Japan) according to the manufacturer’s instructions. Erastin (HY-15763), Ferrostatin-1 (Fer1) (HY-100579) and Acetylcysteine (NAC) (HY-B0215) were purchased from MedChemExpress (MCE, SHH, CHN). SK-HEP1 and SMMC-7721 cells were plated in a 96-well plate with 3000 cells per well and 5 wells as a set and incubated in a humidified cell incubator with an atmosphere of 5% CO2 at 37 °C for 24 h. Then, after treating the cells with each reagent for 72 h, the kit reagent WST-8 was added, and incubation was continued for another 4 h. The OD was measured at 450 nm using SpectraMax iD5(San Jose, CA, USA). Each experiment was repeated at least twice.

### Detection of reactive oxygen species (ROS) accumulation

SK-HEP1 and SMMC-7721 cells were plated in a 6-well plate with 50,000 cells per well and incubated in the humidified cell incubator with an atmosphere of 5% CO2 at 37 °C for 24 h and treated with each reagent for another 48 h. Then DCFH-DA (Invitrogen, CA, USA) was added to the cells and incubated for 30 min, and the ROS accumulation in 10,000 cells was detected by flow cytometry using a microplate reader.

### Iron assay

The iron colorimetric assay kit was purchased from Biovision (Milpitas, California, USA) to measure intracellular iron concentration according to the manufacturer’s instructions. In this assay, ferric iron is dissociated into solution by ferric carrier proteins in an acid buffer environment. The iron content in the sample is measured after iron is reduced to ferrous form (Fe2 +) and reacts with Ferene S to form a stable colored complex.

### IHC staining

The tumor tissues and adjacent non-tumor tissues were fixed in 10% formalin for 1 week and then embedded in paraffin. Four-micrometer sections of tissue specimens were prepared and deparaffinized and antigen was retrieved by microwaving. Immunostaining was performed with following monoclonal antibodies: ABCB6 (Proteintech,51,007–1-AP), FLVCR1 (Abcam, ab251916), SLC48A1 (Novus Biologicals,NBP1–91563) and SLC7A11(Proteintech, 26,864–1-AP).

### Nude mouse xenograft assay

Male BALB/c-nude mice aged 4–6 weeks were purchased from Shanghai Slac Laboratory Animal Co. LTD (Shanghai, China) for the construction of HCC xenograft mouse models. After resuspending in PBS, SMMC-7721 cells (6 × 10 ^ 6/mouse) were injected subcutaneously into the ventral side of nude mice. Nude mice were randomly divided into two groups (5 mice/group) and kept in a sterile environment with 12 h of light/12 h of darkness per day. One week after implantation, when the subcutaneous tumor was visible to the naked eye (approximately 2 mm), mice were treated with 40 mg/kg erastin (intraperitoneal injection, three times a week) or vehicle control (saline). The tumor volume (TV) was calculated according to the following formula: TV (mm3) = L × W ^2^ × 0.5.

### Quantitative real-time polymerase chain reaction (qRT-PCR)

Total RNA was extracted from SK-HEP1 cells and SMMC-7721 cells treated with erastin using TRIzol reagent (Invitrogen, Carlsbad, USA) and reverse transcribed using a cDNA reverse transcription kit (TransGen, Guangzhou, China) in accordance with the manufacturer’s instructions, and the obtained cDNA was amplified using a SYBR Green PCR kit (TransGen, Guangzhou, China). qRT-PCR was performed to detect expression levels in samples. The primers used for qRT-PCR were purchased from TsingKe (Beijing China). Each experiment was repeated three times. The 2-ΔΔCT methodology was adopted to calculate the expression of genes.

### Statistical analysis

Student’s two-sided t-tests in Prism 7.0 (GraphPad, San Diego, CA, USA) were used to compare the differences between two groups. The results are presented as the mean ± standard deviation (SD) of at least five independent experiments. *P* < 0.05 was considered statistically significant.

## Results

### Identification of DEGs related to ferroptosis and iron metabolism in HCC

**Fig. 1:**
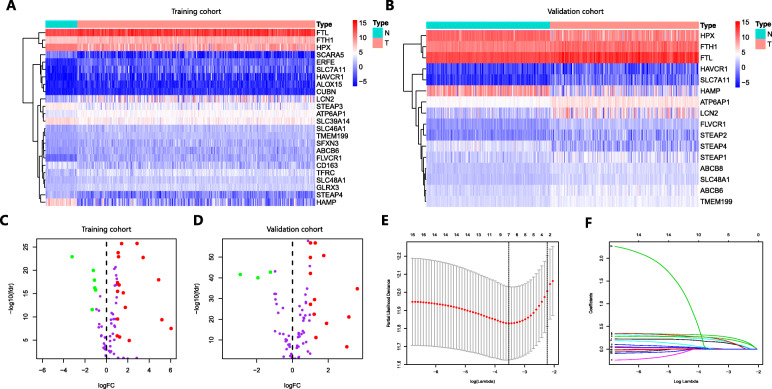
Heatmap, volcano plot and LASSO Cox regression identified the DEGs closely associated with prognosis in HCC. **a** and **c** Gene expression levels in the TCGA database. **b** and **d** Gene expression levels in the ICGC database. **e** and **f** LASSO Cox regression was performed to identify the DEGs closely related to the prognosis of HCC

A total of 104 ferroptosis- and iron metabolism-related genes were identified to match the mRNA-sequencing data in the TCGA and ICGC databases. Using limma with an absolute log2-fold change (FC) > 1 and an adjusted *P* value < 0.05 to perform differential expression analysis, we identified 24 DEGs (17 upregulated and 7 downregulated) in TCGA (Fig. 1a and c) and 16 DEGs (13 upregulated and 3 downregulated) in ICGC (Fig. 1b and d) that were related to ferroptosis and iron metabolism in HCC.

### Comprehensive analysis of the ferroptosis- and iron metabolism-related genes closely associated with prognosis in HCC

We performed univariate Cox regression to explore the relationship between the expression of the 24 DEGs obtained from TCGA and prognosis using 371 HCC samples with OS rates and survival status in TCGA. Sixteen DEGs were statistically significant (*P* < 0.05) and considered to be associated with the prognosis of HCC. Then, LASSO Cox regression was applied to these genes. LASSO is a penalized regression method that adjusts the regression coefficient with L1 penalty to reduce the final weight of most potential indicators to zero, thereby decreasing the number of indicators with a final weight of nonzero [32]. Based on the LASSO regression with 10-fold cross-validation, we screened 7 genes with a repetition frequency greater than 900 times in 1000 substitution samplings (Fig. 1e-f). Matching the 7 genes with 16 DEGs in ICGC, we finally determined that 4 genes (ABCB6, FLVCR1, SLC48A1 and SLC7A11) were significantly associated with prognosis in HCC.

### Building the prognostic signature based on the four ferroptosis- and iron metabolism-related genes and validating its predictive performance

**Fig. 2:**
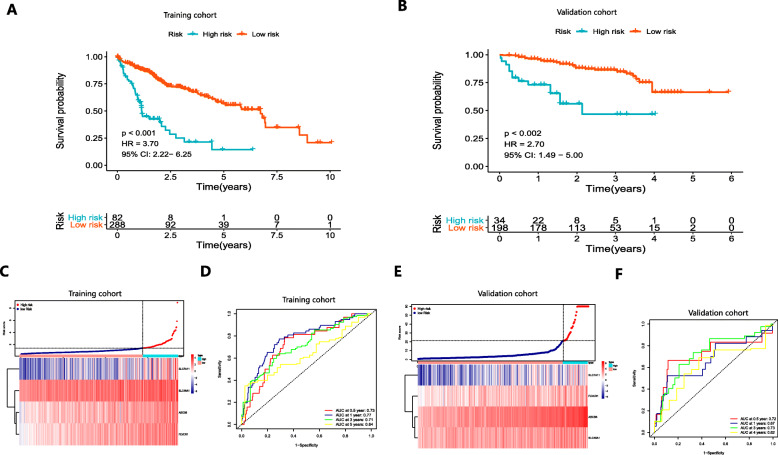
K-M survival analysis, risk score distribution and time-dependent ROC curves of a prognostic model in the HCC cohort from TCGA (**a**, **c**-**d**) and ICGC (**b**, **e-f**). **a** and **b** K-M survival curves indicated that the OS in the high-risk group was markedly poorer than that in the low-risk group (*P* < 0.001). **c** and **e** Distribution of risk scores under different gene expression characteristics in HCC. **d** and **f** Time-dependent ROC curve analysis for measuring the predictive performance on OS

Based on a multivariate Cox regression of the four genes (ABCB6, FLVCR1, SLC48A1 and SLC7A11), we built a prognostic signature. Prognostic index (PI) = (0.135 * expression level of ABCB6) + (0.167 * expression level of FLVCR1) + (0.051 * expression level of SLC48A1) + (0.083 * expression level of SLC7A11). The optimal cut-off value was determined to be 1.4 using X-tile software and performed to divide 370 patients with HCC in the HCC cohort from TCGA into the high-risk and low-risk groups (Figure S1). The underlying diseases of HCC (including viral hepatitis, alcohol consumption, non-alcoholic fatty liver or hemochromatosis) were determined to not affect the expression profiles of these 4 genes in patients (Figure S2). OS was significantly worse in the high-risk groups than that in the low-risk groups (*P* < 0.001, HR = 3.70, 95% CI:2.22–6.25) (Fig. 2a). Figure 2c shows the distribution of risk scores corresponding to gene expression levels. The area under the curve (AUC) in the time-dependent ROC at 0.5, 1, 3 and 5 years reached 0.73, 0.77, 0.71 and 0.64 (Fig. 2d), indicating great specificity and sensitivity of the prognostic signature in predicting OS. We then used the 243 HCC samples in the ICGC to validate the predictive performance of the prognostic signature. PI was calculated according to the formula mentioned earlier, and the optimal cutoff value determined by X-tile software for dividing 243 HCC samples into the high-risk group and low-risk group was 21.3. Consistent with the above results, patients with HCC in the high-risk group had a significantly lower OS than those in the low-risk group (*P* < 0.001, HR = 2.70, 95% CI: 1.49–5.00) (Fig. 2b). The risk score distribution and gene expression are shown in Fig. 2e. The AUCs for 0.5-, 1-, 3- and 5-year OS were 0.72, 0.67, 0.73 and 0.62, respectively (Fig. 2f).

### Construction and validation of the predictive nomogram in the HCC cohort from TCGA

**Fig. 3:**
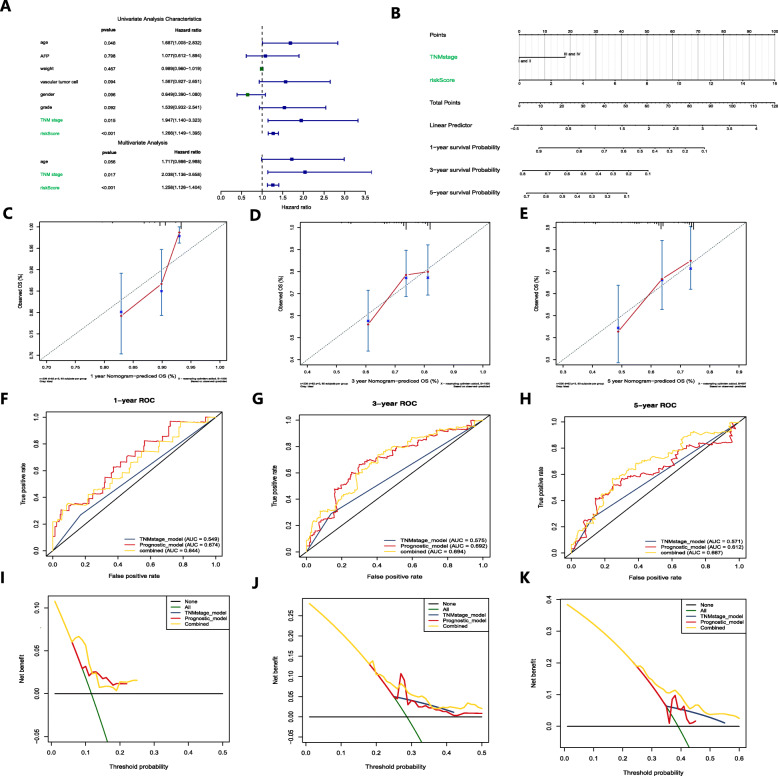
Construction and validation of a predictive nomogram. **a** Univariate and multivariate Cox regression confirmed that the prognostic signature and TNM stage were independent prognostic predictors. **b** The nomogram for predicting the OS of patients with HCC at 1, 3, and 5 years. **c-e** Calibration curves of the nomogram for OS prediction at 1, 3, and 5 years. **f-h** ROC curves to evaluate the predictive ability of the nomogram. **i-k** DCA curves determined that the nomogram can provide optimal clinical decision-making benefits

To determine whether the predictive ability of the prognostic signature in predicting OS was independent of other traditional clinical characteristics (including age, AFP, weight, vascular tumor cell, sex, pathological grade and TNM stage), we performed univariate and multivariate Cox regression analyses on these variables using 370 HCC samples with clinical information in TCGA (Table S1). The results determined that TNM stage (HR = 2.038) and risk score of the prognostic signature (HR = 1.258) were independent predictive factors for predicting OS (Fig. 3a). The proportional hazards of the two independent predictive factors was exhibited in Figure S3. Based on the two independent predictive factors, we constructed a predictive nomogram to quantify the prediction results of individual survival probability at 1, 3 and 5 years (Fig. 3b). The C index for the nomogram was 0.66, with 1000 cycles of bootstrapping (95% CI: 0.55–0.72), and the calibration curves of the nomogram showed great consistency between the predicted OS rates and actual observations at 1, 3 and 5 years (Fig. 3c-e).

We then performed ROC curve analysis to validate the predictive value of the nomogram. The AUCs for 1-, 3- and 5-year OS with the nomogram were 0.644, 0.694 and 0.667, respectively, superior to a single independent predictive factor (Fig. 3f-h). To further determine the value of the nomogram in clinical decision making, we performed DCA. DCA is a new reliable evaluation tool that quantifies the clinical value of a nomogram by analyzing the clinical results obtained from the decision based on the nomogram and has important value in determining the diagnosis and adjusting the prognosis strategy [33]. We found that compared to a single independent predictive factor, the nomogram could obtain the optimal net benefit at 1, 3 and 5 years (Fig. 3 I-J).

### The diagnostic models were established and validated for high specificity and sensitivity

**Fig. 4:**
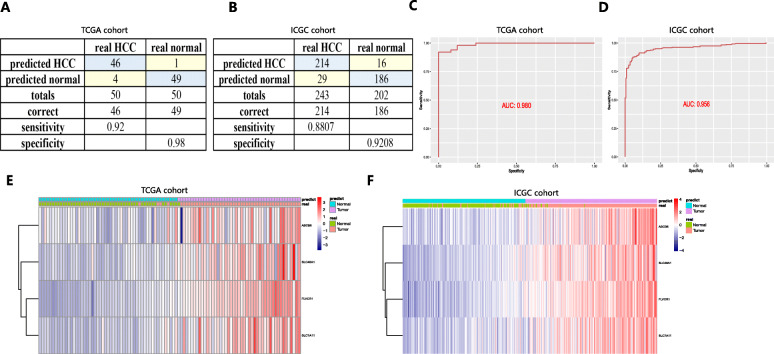
A diagnostic model for distinguishing HCC from normal samples in the HCC cohort from TCGA (**a**, **c** and **e**) and ICGC (**b**, **d** and **f**). **a** and **b** Confusion matrix for the binary classification results of the diagnostic model. **c** and **d** ROC curves for evaluating the predictive performance of the diagnostic model. **e** and **f** Unsupervised hierarchical clustering of the four ferroptosis- and iron metabolism-related genes for the diagnostic model

A diagnostic model integrating the four genes was established to distinguish HCC from normal subjects using a stepwise logistic regression method. Diagnostic scores were identified as follows: logit (*P* = HCC) = − 15.2439 + (− 0.0327 × ABCB6 expression level) + (8.0880 × FLVCR1 expression level) + (3.1229 × SLC48A1 expression level) + (0.1703 × SLC7A11 expression level). Applying the diagnostic model, there was 92.00% sensitivity and 98.00% specificity in the HCC cohort from TCGA (containing 50 normal samples and paired 50 HCC samples) (Fig. 4a) and 88.07% sensitivity and 92.08% specificity in the HCC cohort from ICGC (containing 202 normal samples and 243 HCC samples) (Fig. 4b). ROCs in the HCC cohort from TCGA (AUC = 0.980) (Fig. 4c) and ICGC (AUC = 0.956) (Fig. 4d) were also determined to have great value in accurately distinguishing HCC from normal samples. Unsupervised hierarchical clustering of the four genes indicated a superior ability to differentiate HCC from normal samples (Fig. 4e and f).

**Fig. 5:**
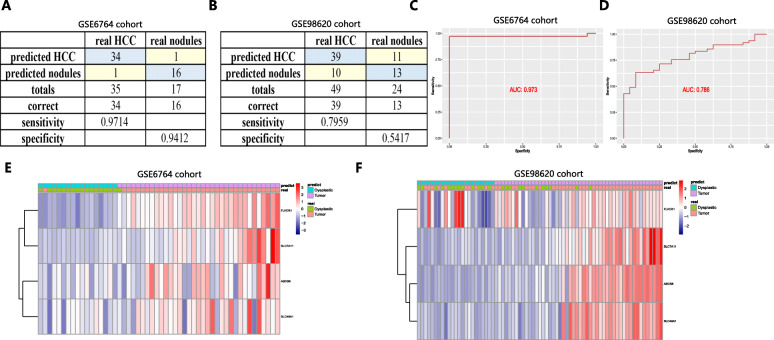
A diagnostic model for distinguishing HCC from dysplastic nodules in the training dataset (GSE6764) (**a**, **c** and **e**) and validation dataset (GSE98620) (B, D and F). **a** and **b** Confusion matrix for the binary classification results of the diagnostic model. **c** and **d** ROC curves for evaluating the predictive performance of the diagnostic model. **e** and **f** Unsupervised hierarchical clustering of the four ferroptosis- and iron metabolism-related genes for the diagnostic model

Since nodules less than 2 cm in the liver were difficult to distinguish from HCC through radiological or pathological examinations [34], we also constructed a diagnostic model based on the four genes in the training cohort (GSE6764) (containing 35 HCC samples and 17 dysplastic nodule samples) for differentiating nodules from HCC samples and validated it in the test cohort (GSE98620) (containing 49 HCC samples and 24 dysplastic nodule samples). Diagnostic scores were identified as follows: logit (P = HCC) = − 13.9106 + (1.3676 × ABCB6 expression level) + (− 0.1018 × FLVCR1 expression level) + (− 0.2817 × SLC48A1 expression level) + (1.1909 × SLC7A11 expression level). The AUCs for the diagnostic model reached 0.973 in the training cohort, with 97.14% sensitivity and 94.12% specificity (Fig. 5a and c), and 0.786 in the test cohort, with 79.59% sensitivity and 54.17% specificity (Fig. 5b and d). Figure 5e and f show unsupervised hierarchical clustering of the four genes.

### Comparison of the immune microenvironment of patients with HCC between the high-risk and low-risk groups

**Fig. 6:**
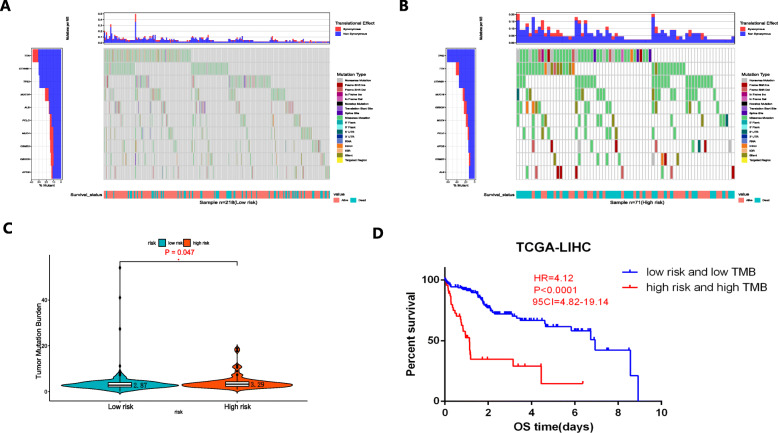
Correlations between risk scores and TMB and the predictive performance of TMB on OS. **a-b** The differences in TMB in somatic cells in patients with HCC between the high- and low-risk groups. **c** The high-risk group showed a higher TMB than the low-risk group. **d** OS rates in patients with low risk and low TMB were higher than those in patients with high risk and high TMB

Since drugs targeting immune checkpoints have been shown to achieve antitumor effects by reversing the immunosuppressive effects of tumors, the expression of immune checkpoints has attracted widespread attention as a biomarker for identifying patients with HCC to receive immunotherapy [35]. The TMB can be used to predict the efficacy of immune checkpoint blockade and has been proven to be a biomarker for identifying patients who can benefit from immunotherapy in several cancer types [36]. In this study, we analyzed the association between risk scores and TMB. Figure 6a and b indicate the differences in TMB in somatic cells in patients with HCC between the high- and low-risk groups. Patients in the high-risk group had a higher TMB than patients in the low-risk group (Fig. 6c). A higher OS rate was obtained in patients with low risk and low TMB group than that in patients with high risk and high TMB group (*P* < 0.0001) (Fig. 6d).

**Fig. 7:**
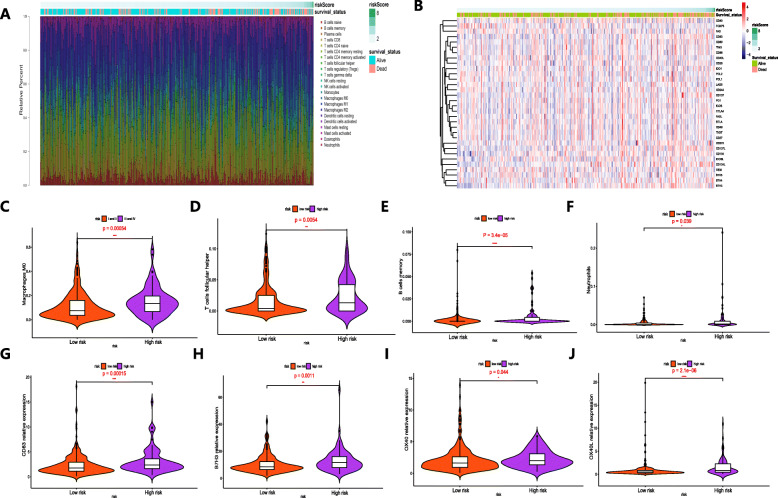
The landscape of immune infiltration and expression of immune checkpoints in patients with HCC with different risk scores. **a** The correlations between risk score and immune infiltration of 22 immune cell types in patients with HCC. **b** The relationship between the risk score and the expression of immune checkpoints. **c-f** Violin plots visualizing fractions of different immune cells in the high-risk and low-risk groups. **g-j** Violin plots visualizing the expression of immune checkpoints in the high-risk and low-risk groups

The differences in immune infiltration of 22 immune cell types obtained from 289 patients with HCC from the TCGA database are shown in Fig. 7a, which may represent an intrinsic feature that can characterize individual differences. Patients with HCC in the high-risk group had higher ratios of M0 macrophages, follicular helper T cells, memory B cells, and neutrophils than those in the low-risk group (*P* < 0.05) (Figs. 7c-f). Figure 7b shows the relationship between the risk score and the expression of immune checkpoints. We found that the expression levels of CD83, B7H3, OX40 and OX40L in the high-risk group were significantly higher than those in the low-risk group (*P* < 0.05) (Fig. 7g-j), suggesting that the poor prognosis of high-risk patients was partly due to the immunosuppressive microenvironment. The results above indicated that abnormal immune infiltration and expression differences of immune checkpoints in HCC can be used as prognostic indicators and targets for immunotherapy, with important clinical significance.

### Internal and external validation of the expression patterns and prognostic predictive performance of the four ferroptosis- and iron metabolism-related genes

**Fig. 8:**
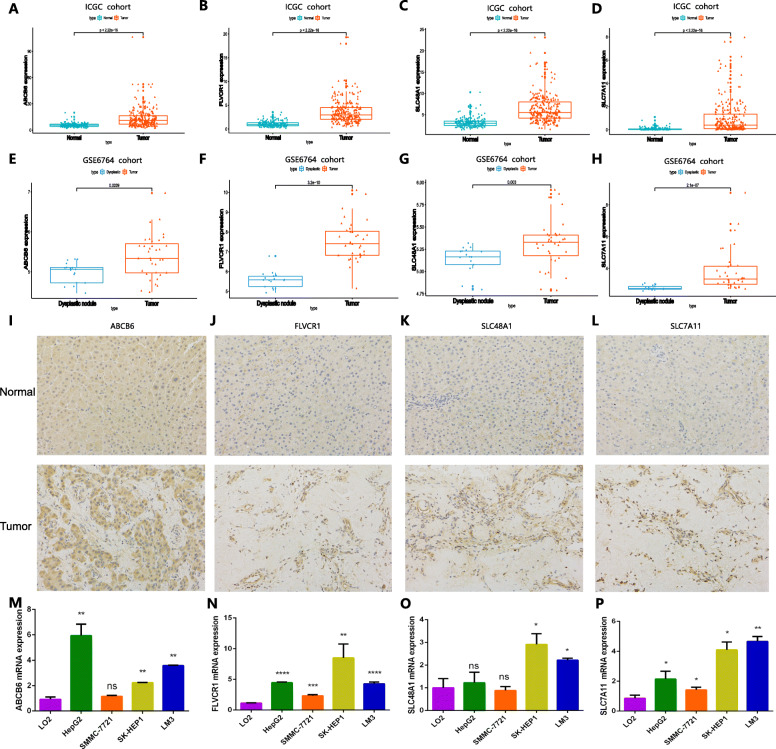
Validation of the expression patterns of the four ferroptosis- and iron metabolism-related genes. **a-d** Expression levels of the four genes in HCC and normal samples in the HCC cohort from ICGC. **e-h** Expression levels of the four genes in HCC and dysplastic nodule samples in the GSE6764 cohort. **i-l** Expression patterns of the four genes in HCC tissues and normal tissues. **m-p** The expression characteristics of the four genes in multiple types of HCC cell lines

The expression levels of ABCB6, FLVCR1, SLC48A1, and SLC7A11 were significantly higher in the HCC cohort from ICGC than in normal samples (*P* < 0.001) (Fig. 8a-d), which was consistent with the predictive analysis of diagnosis and prognosis, demonstrating that the four genes were suitable for constructing diagnostic and prognostic models. For further validation, we detected the expression characteristics of the four genes in the GSE6764 cohort. The four genes presented markedly higher expression in HCC than in dysplastic nodule samples, consistent with the findings above (Fig. 8e-h). Meanwhile, we also evaluated the expression levels of the proteins encoded by these four genes in four pairs of human HCC tissues and corresponding non-tumor tissues to validate the clinical relevance of the four genes. The results of immunohistochemistry (IHC) staining confirmed that ABCB6, FLVCR1, SLC48A1 and SLC7A11 were strongly positive in HCC tissues compared with normal tissues (Fig. 8i-l). In addition, the expression profiles of the four genes in multiple cell lines are shown in Fig. 8m-p.

**Fig. 9:**
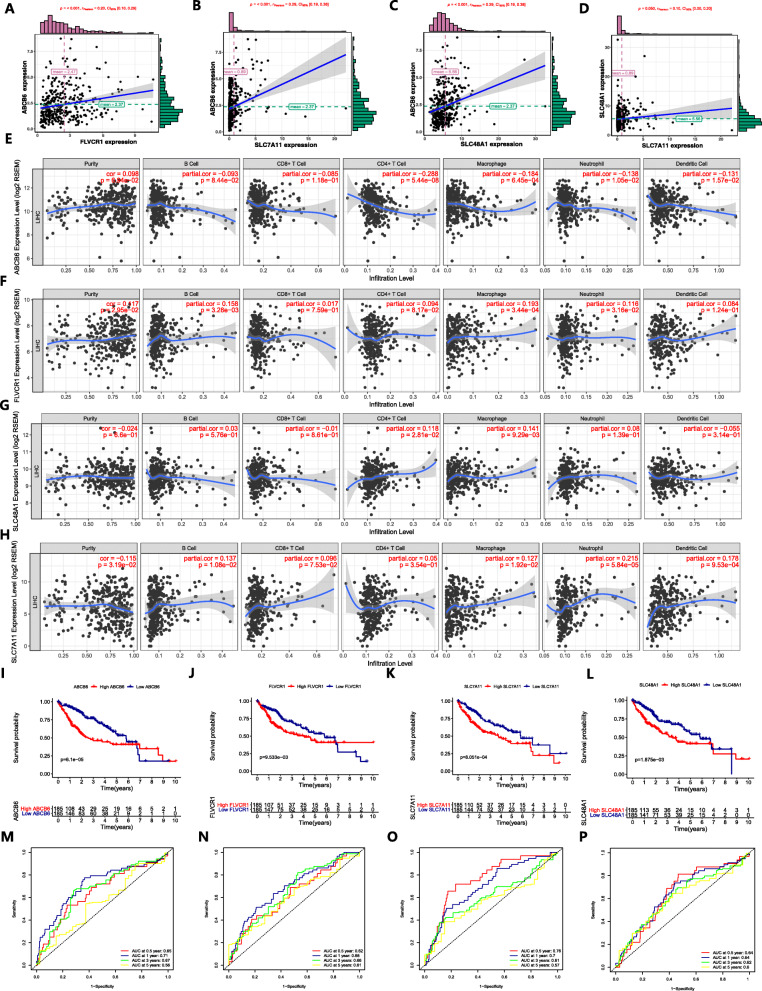
Regression analysis of expression levels among the four genes, correlations between genes and the density of the immune infiltrate, and predictive performance of genes on OS. **a-d** There was a synergistic effect among the expression levels of genes. **e-h** The impact of the expression of ABCB6 (e), FLVCR1 (**f**), SLC48A1 (**g**) and SLC7A11 (**h**) on infiltration by different immune cells. **i-l** K-M survival curves show the OS in the high-expression group and low-expression group. **m-p** Time-dependent ROC curve analysis for evaluating the predictive accuracy of the four genes for 0.5-, 1-, 3- and 5-year OS

Since the four genes exhibited high expression in the tumor tissues, we explored the correlation among the genes. The expression of ABCB6 had synergy with the expression of FLVCR1, as well as the expression of ABCB6 and SLC7A11, ABCB6 and SLC48A1, and SLC48A1 and SLC7A11, which also had the same positive correlation (Fig. 9a-d). The correlation between the expression of the four genes by HCC cells and the immune infiltrate is shown in Fig. 9e-h. K-M curve analysis was performed to validate the predictive value of the four genes in OS. Genes with high expression had lower OS rates than those with low expression (Fig. 9i-l). ROCs validated the predictive performance with high sensitivity and specificity (Fig. 9m-p).

### Inhibition of erastin on the proliferation and tumorigenesis of HCC and its possible molecular mechanism

**Fig. 10:**
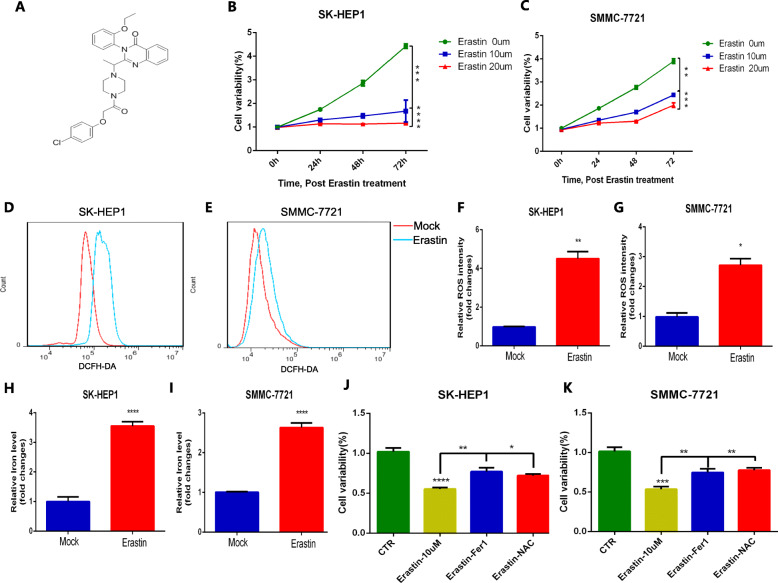
Erastin treatment suppressed proliferation of HCC in vitro. **a** The chemical formula of erastin. **b-c** The CCK-8 assay showed that erastin inhibited the proliferation of SK-HEP1 cells (**a**) and SMMC-7721 cells (**b**) in a dose-dependent manner. **d-g** Erastin stimulation enhanced ROS accumulation in SK-HEP1 cells (**d** and **f**) and SMMC-7721 cells (**e** and **g**). **h**-**i** Erastin treatment increased iron levels in SK-HEP1 cells (**h**) and SMMC-7721 cells (**i**). **j-k** Erastin inhibited the survival and proliferation of SK-HEP1 cells (**j**) and SMMC-7721 cells (**k**) by inducing ferroptosis

**Fig. 11:**
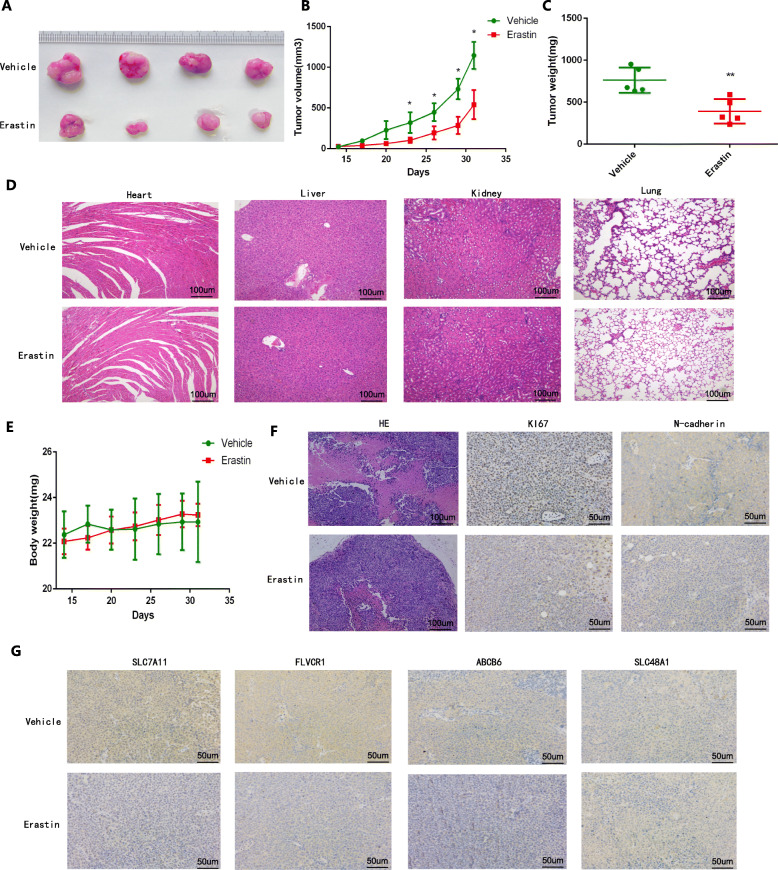
Erastin treatment inhibited tumorgenesis and development of HCC in vivo. a-c The original tumors (**a**), tumor volume (**b**) and tumor weight (**c**) under erastin treatment. **d** Histological changes of heart, liver, lung and kidneys in tumor-bearing and non-bearing mice under elastin treatment. **e** Weight change of mice under erastin treatment over time. **f** Pathological characteristics of tumor tissues and expression of Ki67 and N-cadherin in tumor tissues under erastin treatment. G Expression differences of ABCB6, FLVCR1, SLC7A11 and SLC48A1 between erastin-treated tumor tissues and vehicle-treated tumor tissues

As an inducer of ferroptosis, erastin was used to evaluate its influence on the development and progression of HCC [37]. The chemical formula of erastin was showed in Fig. 10a. Performing the CCK-8 assay, we found that erastin treatment inhibited cell proliferation in a dose-dependent manner (Fig. 10b-c). And we validated that erastin treatment significantly increased the accumulation of ROS (Fig. 10d-g) and iron (Fig. 10h-i) in cells. In addition, Ferrostatin-1 and NAC, regarded as ferroptosis inhibitor and ROS inhibitor, were obviously rescued the anti-proliferation effect of erastin in HCC cells (Fig. 10j-k) indicating that erastin induced ferroptosis to inhibit the proliferation and progression of HCC. To further evaluate the anti-tumoral effect of erastin in vivo, we constructed subcutaneous HCC xenograft models in male BALB/c nude mice by subcutaneous injection of SMMC-7721 cells. Then we treated tumor-bearing mice with erastin and vehicle, respectively. To access the potential toxicity of elastin to organs, we also performed the same elastin treatment on the non-tumor bearing male BALB/c nude mice. Figures 11a-c indicates that erastin significantly inhibits the rate of tumor volume and weight gain in mice. Importantly, we tested important organs (heart, liver, lung and kidneys) in tumor-bearing and non-tumor-bearing mice treated with erastin and confirmed that erastin treatment is nontoxic (Fig. 11d). Compared with vehicle treated tumor-bearing mice, erastin-treated tumor-bearing mice did not undergo significant changes in body weight (Fig. 11e). Lower expression levels of Ki67 and N-cadherin were exhibited in tumor tissues under erastin treatment (Fig. 11f). Moreover, we observed that the expression of ABCB6, FLVCR1 and SLC7A11 in erastin-treated tumor tissues was significantly lower than that in vehicle-treated tumor tissues, but there was no significant difference in the expression of SLC48A1 between the two groups (Fig. 11g).

**Fig. 12:**
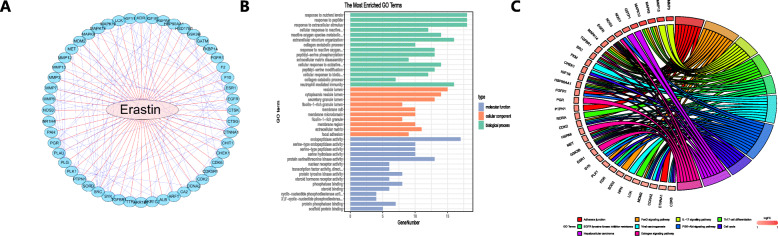
Possible molecular mechanism by which erastin inhibits the proliferation and progression of HCC. **a** The interaction of proteins regulated by erastin. **b**-**c** GO analysis (**b**) and KEGG pathway analysis (**c**) of the potential targets of erastin

As it was determined that erastin inhibited the proliferation and progression of HCC, we explored the possible molecular mechanism by which erastin achieves antitumor effects. In the Cancer Therapeutics Response Portal (CTRP) database (http://portals.broadinstitute.org/ctrp/), 52 genes were shown to be regulated by erastin, and their association is exhibited in Fig. 12a. By performing Gene Ontology (GO) (Fig. 12b) and Kyoto Encyclopedia of Genes and Genomes (KEGG) pathway enrichment analyses (Fig. 12c) on these genes, we found that erastin could cause changes in signaling cascades, including Th17 cell differentiation and the IL-17 signaling pathway (*P* < 0.05). This result indicated that the IL-17 signaling pathway is a potential target affected by erastin in this study.

## Discussion

As a major leading cause of cancer-related mortality worldwide, HCC presents a major health burden for society [38]. Among the current multiple treatments, liver transplantation and tumor ablation are still the only options that may lead to a cure [39]. However, most patients are diagnosed at an advanced stage, and these treatments cannot be selected. The 5-year recurrence rate is very high even in patients who have received liver resection or liver transplantation [40, 41], and the 5-year survival rate is still low [42]. Since HCC is a molecular heterogeneous malignant tumor, its molecular characteristics are related to corresponding biological behaviors, including cell regeneration, microvascular invasion, and distant metastasis [43], and play an important role in the prognosis of HCC. Therefore, it is necessary to identify key molecular markers that affect the prognosis of HCC, thereby optimizing the early diagnosis of HCC and strengthening treatment to improve the clinical outcome of HCC.

The development of high-throughput array technology provides an opportunity to explore novel genes involved in the occurrence and progression of HCC [44]. Ferroptosis is a regulated autophagic cell death process in which iron-dependent oxidation plays a key role [45]. Disturbances in iron metabolism cause excessive intracellular iron storage and may induce ferroptosis [46]. Ferroptosis is regulated by several genes [47]. Previous studies have confirmed that ferroptosis is an effective mechanism for inducing HCC cell death, but its specific molecular changes and mechanism of action are not fully understood [48, 49].

In this study, we aimed to analyze HCC-related RNA sequences obtained through high-throughput array technology using Cox proportional hazards regression and LASSO methods to determine ferroptosis- and iron metabolism-related genes that were associated with the prognosis of HCC. We found that the prognosis model constructed by four genes (ABCB6, FLVCR1, SLC48A1 and SLC7A11) independently predicted the prognosis of patients with HCC with superior prediction performance. And the expression characteristics of the four genes are not affected by the differences in the underlying diseases of HCC, suggesting that the constructed prognosis model can be applied to various types of HCC patients to predict prognosis. Besides, the corresponding nomogram based on the four-gene model also helps clinicians make better clinical decisions and develop treatment strategies. And the diagnostic models integrating the four genes were useful for the early diagnosis of HCC with high specificity and sensitivity. ABCB6 belongs to the B subfamily of ABC transporters, which is a porphyrin energy-dependent transporter [50]. Gene expression analysis and animal experiments show that loss of Abcb6 can cause up-regulation of heme and iron pathways crucial for normal development [51]. ABCB6 expression plays an important role in the coordination of iron homeostasis [52]. And previous studies have reported a correlation between hepatitis C virus-associated hepatocellular carcinoma and increased ABCB6 mRNA levels. ABCB6 mRNA and DNA methylation levels help predict early intrahepatic recurrence [53]. Feline leukemia virus subgroup C receptor 1, encoded by FLVCR1, plays an important role in iron metabolism, participating in the outflow of iron metabolism, preventing oxidative damage caused by excessive iron [54]. Clinical analysis found that FLVCR1 was significantly negatively correlated with maternal iron levels and placental iron concentration, suggesting that FLVCR1 is essential for iron homeostasis and iron metabolism [55]. Studies also found that FLVCR1 expression is correlated with the prognosis of HCC [56]. SLC48A1 is an endosomal heme transporter that participates in the process of heme iron transport in iron metabolism [57]. Lipid peroxide is triggered by lipid peroxidation, and this process is strictly regulated by SLC7A11 (a key component of the cystine-glutamate antiporter); when lipid peroxide is excessively accumulated, ferroptosis can be induced [58, 59]. It was reported that the expression of SLC7A11 is related to the prognosis of HCC [60]. For evaluating the correlation of these four genes with ferroptosis and iron metabolism, we detected in this study that the encoded proteins of the four genes were strongly positive in the tumor tissues of HCC patients, and further validated in the mouse models that expression of ABCB6, FLVCR1 and SLC7A11 were down-regulated in tumor tissues treated by ferroptosis inducer erastin.

Immunotherapy is a tumor treatment method that uses the body’s own immune system to produce an antitumor response [61]. In order to avoid the antitumor immune response during the development of many types of tumors, immunosuppressive mechanisms will be initiated, and with increased immunosuppressive cells and immunosuppressive molecules, low-immunogenic cancer cells will be selected and an immunosuppressive network (immune escape) will be established [62]. By blocking immunosuppressive mechanisms and the function of immunosuppressive cells, potential antitumor immune responses can be triggered [63]. In recent years, manipulation of immune checkpoints or pathways has become an important and effective form of immunotherapy [61], and high TMB has been identified to correlate with good outcomes of immune checkpoint inhibitor treatments [64]. In this study, we found that patients with HCC with high risk scores identified by the ferroptosis and iron metabolism signatures had higher TMB levels and higher proportions of M0 macrophages, follicular helper T cells, memory B cells and neutrophils, confirming that ferroptosis and iron metabolism have a regulatory effect on the TIME, and also may indicate that the poor prognosis in the high-risk group may be due to a stronger immunosuppressive effect. When detecting immune checkpoints, higher expression of CD83, B7H3, OX40 and OX40L was exhibited in the high-risk group. These differences promote the growth and progression of HCC, leading to a poor prognosis for HCC. In addition, the findings above suggest that patients in the high-risk group may benefit more from immune checkpoint inhibitor therapy than patients in the low-risk group. This provides new insight for tumor immunotherapy.

Erastin has been determined to be an inducer of ferroptosis in previous studies and has been identified as an inhibitor of cystine/glutamate antiporter (xCT) and glutathione synthesis [65, 66]. In this study, we found that erastin has an antitumor effect by inducing ferroptosis to inhibit the proliferation and progression of HCC. Erastin treatment has been shown to inhibit tumor growth in mouse tumor models, which provides new ideas for the treatment of HCC. In addition, erastin could change TH17 cell differentiation and the IL-17 signaling pathway by bioinformatics analysis. IL-17 is a universal cytokine in the tumor microenvironment. In existing tumors, IL-17 achieves an antitumor effect by activating immune cells and inducing indirect immunity [66, 67]. The regulatory potential of the IL-17 immune axis makes IL-17 a compelling target in cancer immunotherapy. These results suggest that the ferroptosis inducer erastin may be regarded as a potential agent of cancer immunotherapy.

## Conclusion

In summary, we identified four ferroptosis- and iron metabolism-related genes with great predictive value in the OS of HCC, and the prognostic and diagnostic models based on the four genes indicated superior diagnostic and predictive performance. As an inducer of ferroptosis, erastin showed an antitumor effect by inhibiting the proliferation and progression of HCC. Through bioinformatics analysis, erastin was shown to affect TH17 cell differentiation and the IL-17 signaling pathway, indicating that it is a potential targeted drug for immunotherapy.

## Supplementary information

**Additional file 1: Figure S1.** The analysis process of X-tile software for determining the optimal cutoff value to divide HCC patients into high prognostic risk group and low prognostic risk group.

**Additional file 2: Figure S2.** The influence of the underlying disease of HCC on the expression of ABCB6, FLVCR1, SLC7A11 and SLC48A1 in patients.

**Additional file 3: Figure S3.** proportional hazards of TNM staging and the prognosis signature over time. A Proportional hazards of TNM staging. B Proportional hazards of the prognostic signature.

**Additional file 4 Table S1.** Univariate and multivariate Cox regression analyses of the prognostic signature and clinical features related to OS in HCC patients.

## Data Availability

The data and materials used to support the findings of this study are available from the corresponding author upon request.

## Abbreviations

HCC: Hepatocellular carcinoma
LASSO: The least absolute shrinkage and selection operator
OS: Overall survival
TMB: Tumor mutation burden
DEG: Differentially expressed gene
HR: Hazard ratio
CI: Confidence intervals
TCGA: The Cancer Genome Atlas
ICGC: International Cancer Genome Consortium
KEGG: Kyoto Encyclopedia of Genes and Genomes
GO: Gene Ontology
ABCB6: ATP binding cassette subfamily B member 6
FLVCR1: FLVCR heme transporter 1
SLC48A1: Solute carrier family 48 member 1
SLC7A11: Solute carrier family 7 member 11
ROC: Receiver operator characteristic curve
AUC: Area Under Curve
DCA: Decision Curve Analysis
CCK-8 assay: Cell Counting Kit-8 Assay
DMEM: Dulbecco Modified Eagle Medium
TIME: Tumor immune microenvironment
