# Supplementary figures and images for: The ferroptosis and iron-metabolism signature robustly predicts clinical diagnosis, prognosis and immune microenvironment for hepatocellular carcinoma
Source: Cell Commun Signal. 2020 Oct 28;18:174. doi: 10.1186/s12964-020-00663-1 (PMC7592541; doi:10.1186/s12964-020-00663-1)

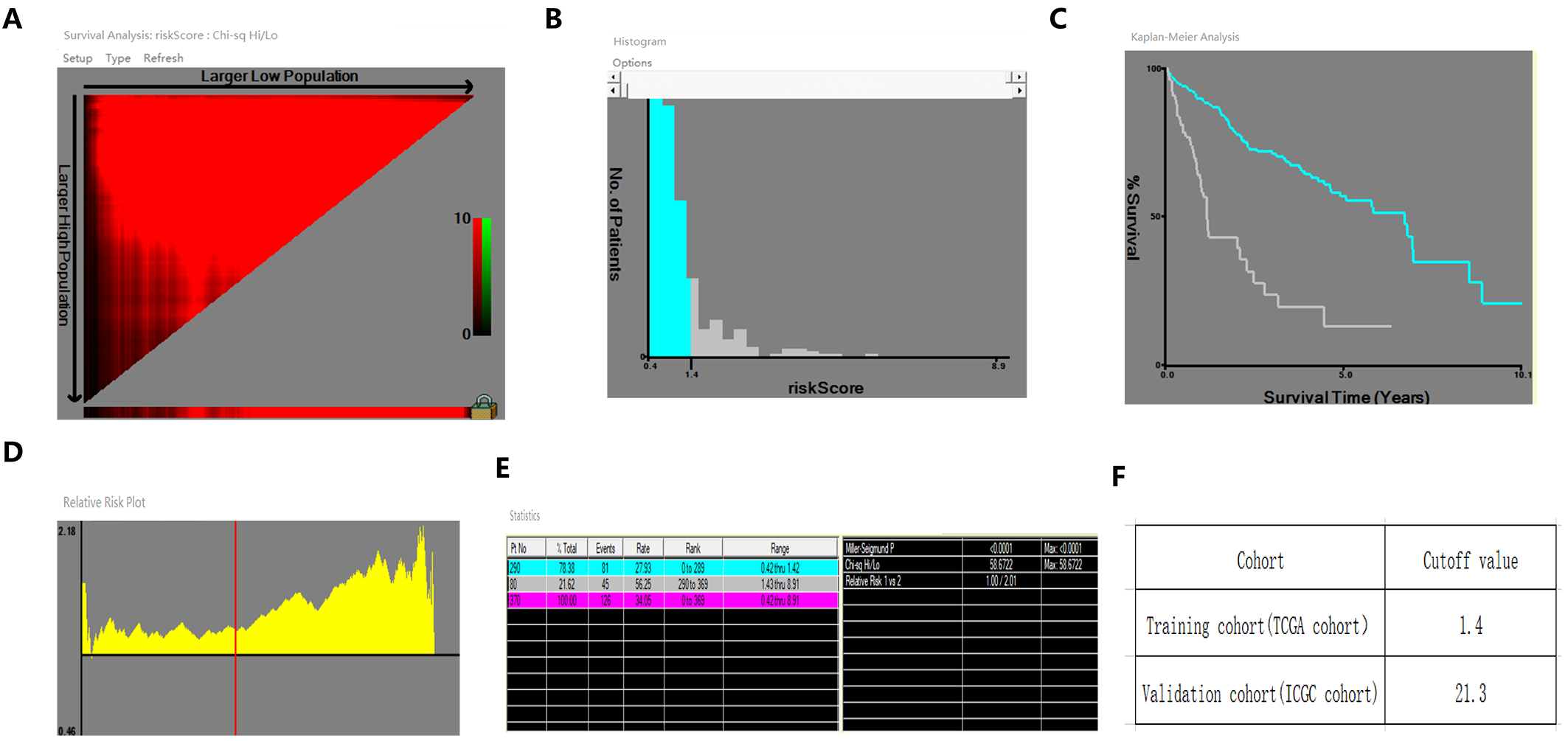

Supplement: Supplementary file 2 — Additional file 1: Figure S1. The analysis process of X-tile software for determining the optimal cutoff value to divide HCC patients into high prognostic risk group and low prognostic risk group. [file 12964_2020_663_MOESM2_ESM.tif]

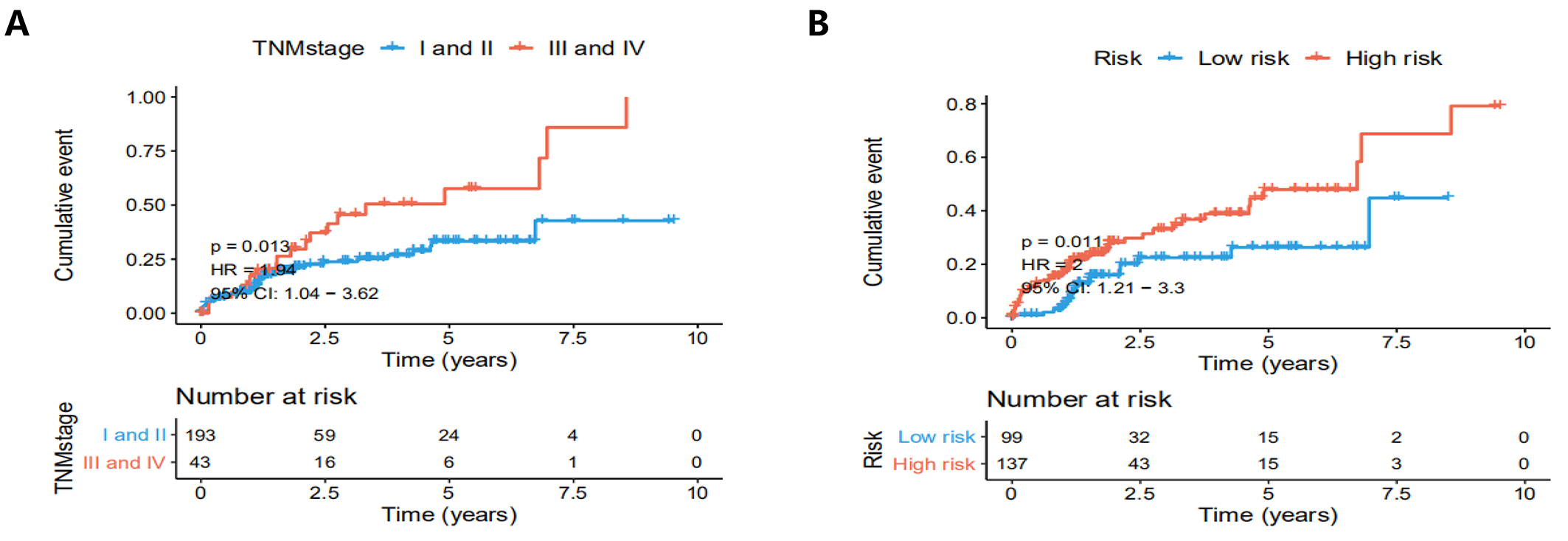

Supplement: Supplementary file 4 — Additional file 3: Figure S3. proportional hazards of TNM staging and the prognosis signature over time. A Proportional hazards of TNM staging. B Proportional hazards of the prognostic signature. [file 12964_2020_663_MOESM4_ESM.tif]
