# Supplementary material for: The ferroptosis and iron-metabolism signature robustly predicts clinical diagnosis, prognosis and immune microenvironment for hepatocellular carcinoma
Source: Cell Commun Signal. 2020 Oct 28;18:174. doi: 10.1186/s12964-020-00663-1 (PMC7592541; doi:10.1186/s12964-020-00663-1)
Supplement: Supplementary file 5 — Additional file 4 Table S1. Univariate and multivariate Cox regression analyses of the prognostic signature and clinical features related to OS in HCC patients. [file 12964_2020_663_MOESM5_ESM.docx]

Table S1: Univariate and multivariate Cox regression analyses of the prognostic signature and clinical features related to OS in HCC patients.

| Characteristics | Univariate analysis | | Multivariate analysis | |
| --- | --- | --- | --- | --- |
|  | HR (95%CI) | P-value | HR (95%CI) | P-value |
| age（≥60/<60） | 1.686576 | 0.048041 | 1.716688 | 0.056054 |
| gender(Male/Female) | 0.649394 | 0.096388 |  |  |
| weight(64-189) | 0.988892 | 0.466736 |  |  |
| vascular tumor cell(yes/no) | 1.567373 | 0.093812 |  |  |
| AFP(≥300/<300) | 1.076633 | 0.797748 |  |  |
| Histologic grade(G3+G4/G1+G2) | 1.539132 | 0.091867 |  |  |
| TNM stage(III+IV/I+II) | 1.946615 | 0.014638 | 2.0384648 | 0.016952 |
| riskScore(high risk group/low risk group) | 1.266445 | 0.000002 | 1.2575550 | 0.000046 |
